# Whole Genome Sequencing Shows a Low Proportion of Tuberculosis Disease Is Attributable to Known Close Contacts in Rural Malawi

**DOI:** 10.1371/journal.pone.0132840

**Published:** 2015-07-16

**Authors:** Judith R. Glynn, José Afonso Guerra-Assunção, Rein M. G. J. Houben, Lifted Sichali, Themba Mzembe, Lorrain K. Mwaungulu, J. Nimrod Mwaungulu, Ruth McNerney, Palwasha Khan, Julian Parkhill, Amelia C. Crampin, Taane G. Clark

**Affiliations:** 1 Faculty of Epidemiology and Population Health, London School of Hygiene & Tropical Medicine, London, United Kingdom; 2 Karonga Prevention Study, Chilumba, Malawi; 3 Faculty of Infectious and Tropical Diseases, London School of Hygiene & Tropical Medicine, London, United Kingdom; 4 Wellcome Trust Sanger Institute, Hinxton, United Kingdom; Fundació Institut d’Investigació en Ciències de la Salut Germans Trias i Pujol. Universitat Autònoma de Barcelona, SPAIN

## Abstract

**Background:**

The proportion of tuberculosis attributable to transmission from close contacts is not well known. Comparison of the genome of strains from index patients and prior contacts allows transmission to be confirmed or excluded.

**Methods:**

In Karonga District, Malawi, all tuberculosis patients are asked about prior contact with others with tuberculosis. All available strains from culture-positive patients were sequenced. Up to 10 single nucleotide polymorphisms between index patients and their prior contacts were allowed for confirmation, and ≥ 100 for exclusion. The population attributable fraction was estimated from the proportion of confirmed transmissions and the proportion of patients with contacts.

**Results:**

From 1997–2010 there were 1907 new culture-confirmed tuberculosis patients, of whom 32% reported at least one family contact and an additional 11% had at least one other contact; 60% of contacts had smear-positive disease. Among case-contact pairs with sequences available, transmission was confirmed from 38% (62/163) smear-positive prior contacts and 0/17 smear-negative prior contacts. Confirmed transmission was more common in those related to the prior contact (42.4%, 56/132) than in non-relatives (19.4%, 6/31, *p* = 0.02), and in those with more intense contact, to younger index cases, and in more recent years. The proportion of tuberculosis attributable to known contacts was estimated to be 9.4% overall.

**Conclusions:**

In this population known contacts only explained a small proportion of tuberculosis cases. Even those with a prior family contact with smear positive tuberculosis were more likely to have acquired their infection elsewhere.

## Background

Understanding where *M*.*tuberculosis* transmission is occurring is key to tuberculosis control. Close contact with someone with tuberculosis is a known risk factor for infection and hence disease, so contact tracing is often recommended.[1,2] Current WHO guidelines reiterate as a “strong recommendation” that household and other close contacts should be actively screened for tuberculosis, but acknowledge that this is based on very low quality evidence.[3]

The proportion of disease due to household and close contacts is not certain, particularly in high prevalence areas. It can be investigated using traditional epidemiological techniques, comparing the contact histories of cases of tuberculosis and controls,[4] but this assumes that the increased risk in the cases is attributable to the contacts rather than shared risk factors, which can be difficult to adjust for. Older DNA fingerprinting techniques such as RFLP or MIRU-VNTR have improved on this by ensuring that the contacts share fingerprint patterns,[5–10] but the level of discrimination is limited if some DNA fingerprint strains are common, and it is impossible to exclude a common source.

Whole genome sequencing allows a more accurate approach:[11–13] the combination of a history of contact and a small number of single nucleotide polymorphisms (SNPs) between the strain in the index patient and their prior contact makes transmission highly likely, and can allow pinpointing of the most likely source if there is more than one. If the proportion of tuberculosis patients with contacts of different types is also known, an accurate estimate can be made of the proportion of tuberculosis attributable to these contacts.

In Karonga District, Malawi, we have been collecting data on all tuberculosis patients and their prior contacts since 1997. We have now used whole genome sequencing on all available case-contact pairs to improve understanding of transmission.

## Methods

The Karonga Prevention study in northern Malawi has been studying tuberculosis in the whole of Karonga district (current population approximately 300,000) since the 1980s. Cases are identified through enhanced passive case finding with project staff based at the district hospital and major health centres to identify those with chronic cough or other symptoms suggestive of tuberculosis. Each patient is interviewed and at least three sputum specimens are taken at diagnosis, with further specimens at follow-up and at the end of treatment. Cultures from sputum (and other specimens if indicated) are set up in the laboratories in Malawi. Those that resemble *M*.*tuberculosis* macroscopically are sent to the UK Mycobacterium Reference Laboratory for species identification and drug resistance testing.

Since March 1997 we have asked all patients about prior contacts with tuberculosis, in their family or household (ever), or other contacts (within the past 5 years). If they report contacts we ask further detail including the duration and location of contact, and whether they had contact when the first case was ill. If these prior contacts were treated within the district they will already be known to us, allowing us to confirm the type of tuberculosis (smear positive, smear negative pulmonary, or extrapulmonary) and other details.

Approval for the study was given by the ethics committee of the London School of Hygiene & Tropical Medicine (#5067) and the Malawian National Health Sciences Research Committee (#424). Informed consent was obtained from all participants, including separate counselling and consent for HIV testing. In the earlier years of the study this was verbal, consistent with practice at that time, with refusals recorded in hand-written registers. Since 2001 written consent has been used.

### SNP differences

We carried out whole genome sequencing of all available cultures from case-prior contact pairs to establish whether transmission can be confirmed or not. The sequencing was carried out at the Sanger Institute, using Illumina HiSeq 2000 technology with paired-end reads of length 100 base-pairs. We used trimmomatic software (http://www.usadellab.org/cms/?page=trimmomatic) to remove low-quality reads and reads <50 base-pairs long. We mapped reads to the H37Rv reference genome (Genbank assession: AL123456.3), using the BWA-mem algorithm (http://bio-bwa.sourceforge.net/) and excluded samples with an average genomic coverage <10-fold.[14]

We identified SNP positions using SAMtools (http://samtools.sourceforge.net/). If alleles at a position were not identical we took the majority allele if it had a frequency of ≥ 75% and the position was supported by ≥20-fold coverage; otherwise we classified the position as missing (thus ignoring heterozygous calls). We excluded samples with >15% missing genotype calls, to remove possible contaminated or mixed samples or technical errors. (The proportion of mixed strains is low in this setting.[15]) We excluded genome positions with >15% missing genotypes, and those in highly repetitive and variable regions (e.g. PE/PPE genes). This quality control left 94% of the *M*.*tuberculosis* genome to be analysed for variants. Median coverage was 88-fold, mean 127. Spoligotyping was performed in silico using SpolPred.[16] Lineages were defined from spoligotype families.[17] We calculated SNP distances between sequences using the ape library in the R statistical package (http://cran.r-project.org/).

Based on the number of SNPs between samples in patients with multiple isolates,[18] and between patients with likely transmission in other analyses[11–13] and in this dataset (see below), we made the following rules: 0–10 SNPs transmission is likely; 11–99 SNPs transmission uncertain; ≥ 100 SNPs no transmission. To ensure that the changes we were measuring were not due to selection pressure from treatment, we reran the analysis excluding positions known to be associated with drug resistance mutations [19]

### Confirming transmission

We excluded index cases with a history of previous tuberculosis, and prior contacts with extrapulmonary tuberculosis (since this is not infectious). For those with more than one contact we selected the most likely source as the one with fewest SNPs different (and then the closest family relation if there was still more than one). Index case-prior contact pairs with ≤10 SNPs were taken as confirmed. The mutation rate in these pairs was estimated using linear regression.

Cases could only be included in the analysis if sequence was available for at least one contact, so those who named more contacts were more likely to be included. However, assuming that tuberculosis is acquired from one source, those with more contacts are less likely to have transmission confirmed from each one. This could underestimate the proportion of confirmed transmissions. We attempted to minimise this bias by selecting the closest contact, as above, and assessed the extent of any remaining bias by comparing the proportion of confirmed transmissions among those with one or more than one named contact.

### Contact analysis

We analysed risk factors associated with confirmation of transmission from the identified contact using logistic regression, after excluding pairs with 11–99 SNPs, and taking those with 0–10 SNPs as confirmed. Risk factors included: characteristics of the index case and the contact (age, sex, HIV status); characteristics of the strain (isoniazid resistance, *M*.*tuberculosis* lineage); and characteristics of the contact (relationship, intensity of contact, and time interval between the case and the contact). Intensity of contact was defined as high if the contact was prolonged, indoors and on more than one day, and very high if the case had nursed the prior contact while they were ill.

### Proportion of cases due to transmission from named contacts

The proportion of confirmed transmissions in the case-contact pairs is the attributable risk percent since it is the proportion of tuberculosis cases with named contacts who have acquired tuberculosis from that contact. To estimate the proportion of tuberculosis cases due to transmission from named contacts in the whole population (the population attributable fraction, PAF) the attributable risk percent was multiplied by the proportion of all new culture-confirmed cases naming at least one contact, and the proportion of named contacts who are smear positive.

Sequence data are available from the European Nucleotide Archive http://www.ebi.ac.uk/ena/data/view/ERP000436 and http://www.ebi.ac.uk/ena/data/view/ERP001072)

## Results

**Fig 1:**
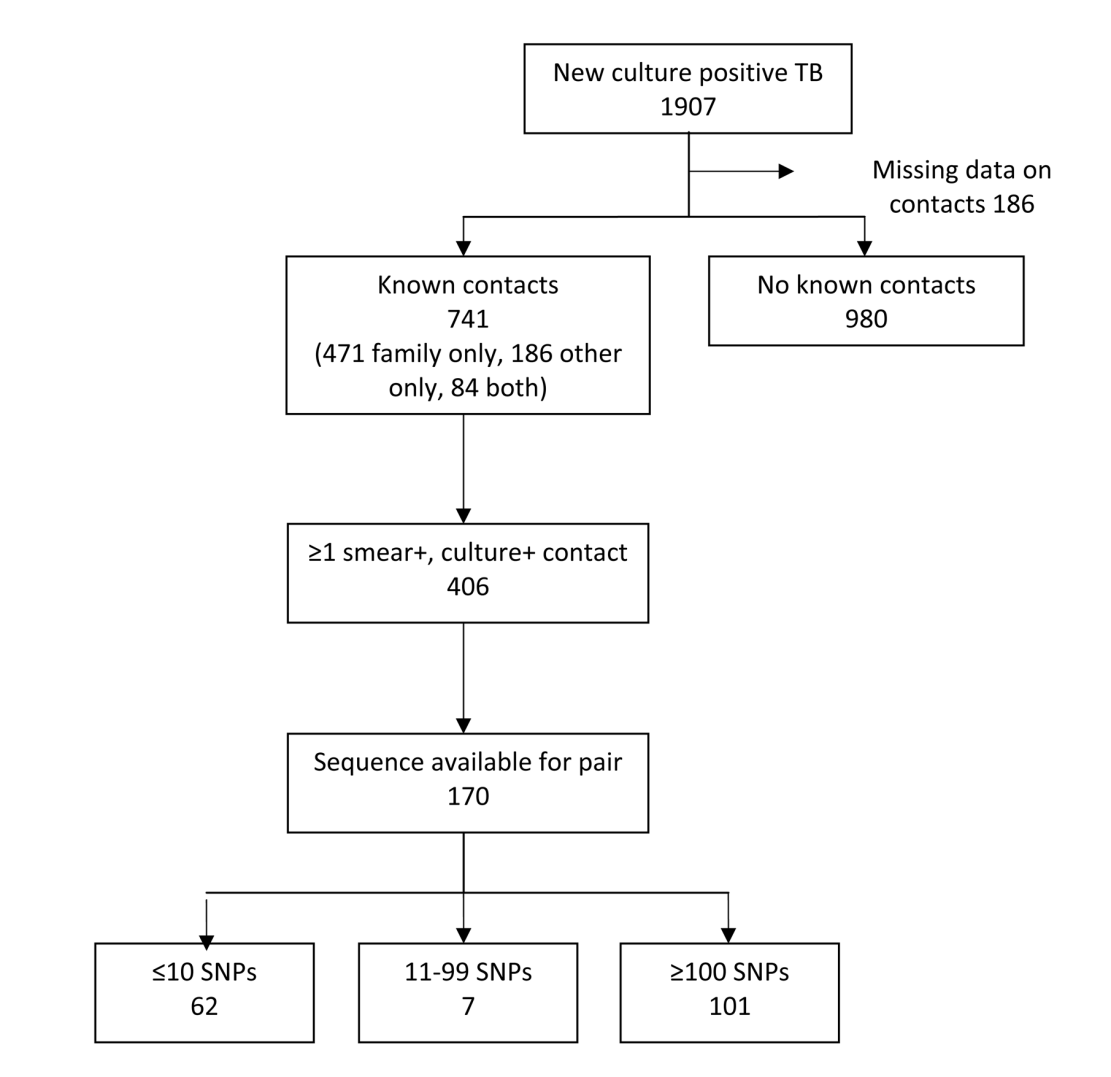
Flow-chart of patients included in the study.

Between March 1997 and March 2010 there were 1907 patients with culture confirmed tuberculosis having their first episode of tuberculosis. As shown in Fig 1, information on prior contacts with tuberculosis was available for 90% (1721/1907): 32.1% (555 of 1727 with recorded data for this variable) had had at least one previous tuberculosis case in their household or close family and 15.8% (270 of1705 with recorded data for this variable) had other known contacts with tuberculosis. In our whole database, of the named contacts reported by tuberculosis patients to have been treated in Karonga District since 1986, 82.0% (1463/1793) were identified in the database as tuberculosis cases, of whom 59.7% (873/1463) had confirmed smear positive pulmonary disease.

**Fig 2:**
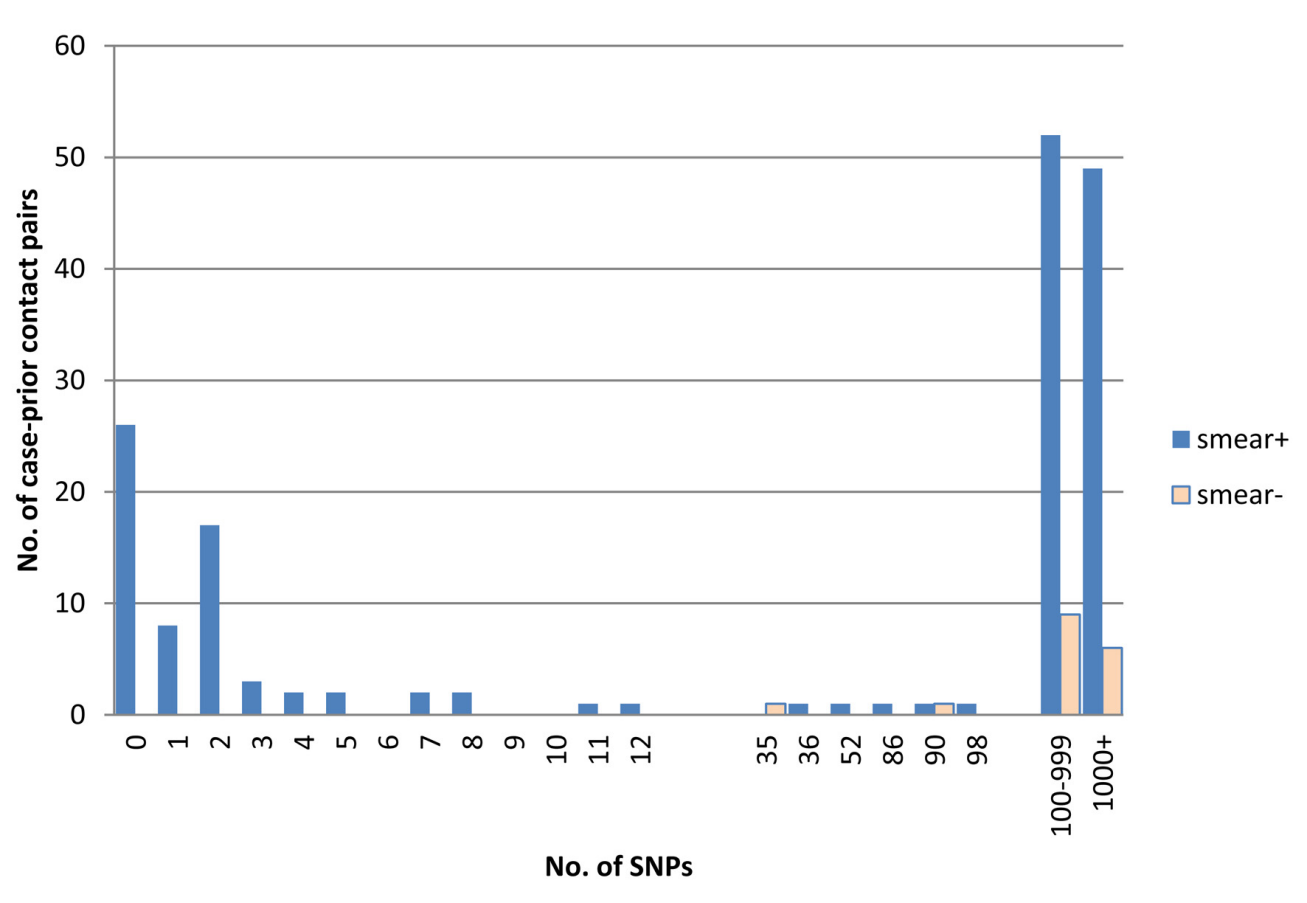
Number of SNPs between index patients and identified prior contacts, by sputum smear status of the prior contact.

Whole genome sequences that passed the quality control were available for the index case and at least one prior contact for 187 index cases; 20 cases had two prior contacts with sequences available. After selecting the most likely source for these 20 cases, there were 187 pairs (170 with a smear positive prior contact and 17 with a smear negative prior contact). The number of SNPs between the 187 pairs is shown in Fig 2: 62 had ≤10 SNPs, 9 had 10–99 SNPs and 116 had ≥100 SNPs. These groups were unchanged by excluding positions associated with drug resistance. Since there was no confirmed transmission from smear negative prior contacts (the minimum SNP distance was 35) the remaining analysis was restricted to smear positive prior contacts.

In the whole dataset over the period of the study there were 406 culture positive index cases with at least one prior culture-confirmed smear positive contact. The 170 included cases were similar to the 236 without paired sequences available in terms of age (mean 36 years for both), sex (41% vs 47% male) and intensity of contact (54% vs 55% low intensity), but there was a difference by calendar year: only 22% of those diagnosed before 2000 were included compared to 55% later.

Of the 170 included pairs, 36% (62/170) had transmission confirmed based on SNPs, or 38% (62/163) after excluding the 7 pairs with uncertain transmission (11–99 SNPS, Fig 1). Among the 163 cases, 83 had named one (identified) contact, and 80 more than one (although we only had the paired sample for more than one contact for 20 of these). The proportion with confirmed transmission was 35% in those with one contact and 41% in those with more than one contact, showing that the failure to include all of the contacts for those with more than one does not appear to have reduced the proportion of confirmed transmissions for this group.

**Fig 3:**
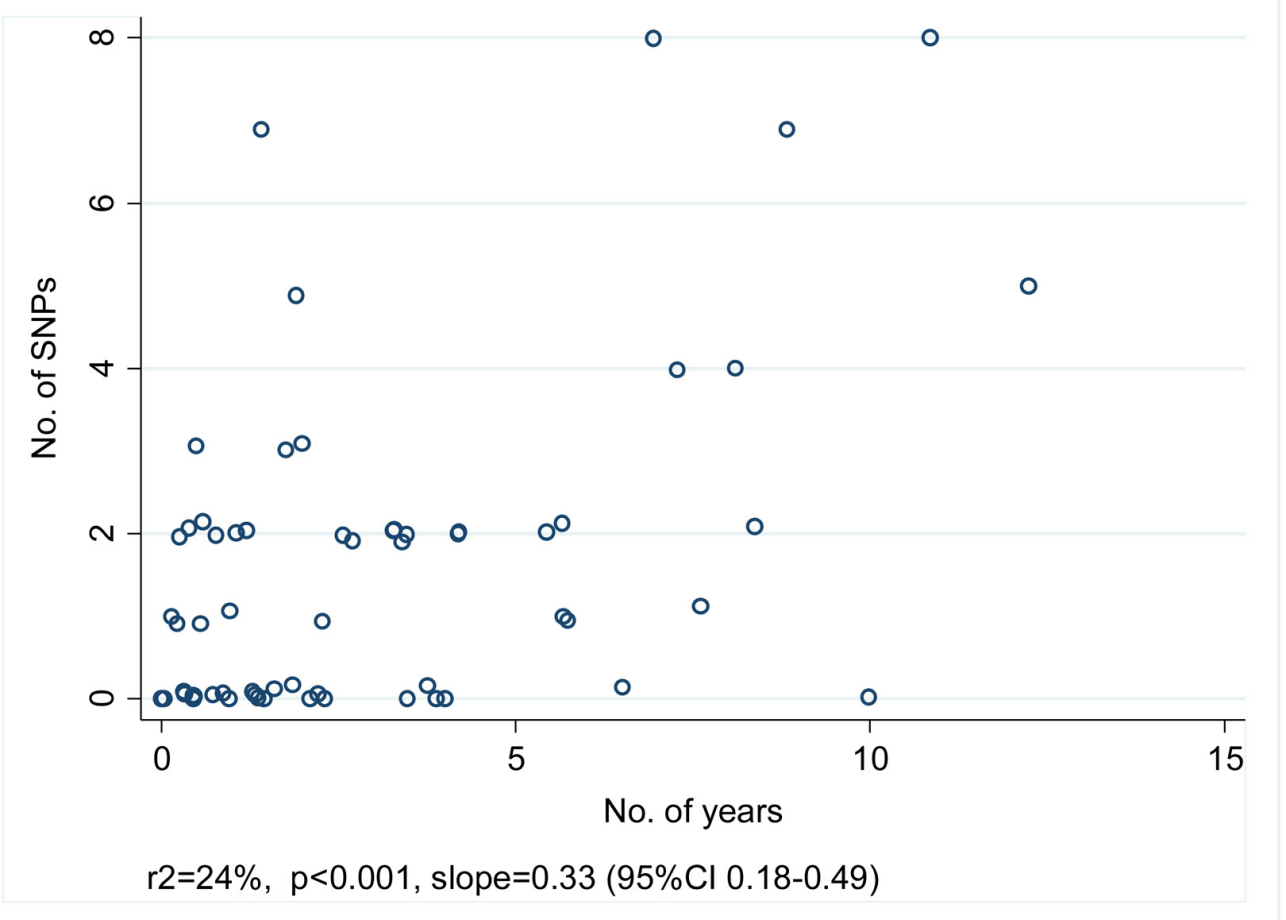
Number of SNPs by time interval between successive cases in 62 case-contact pairs with confirmed transmission. (Random noise has been introduced to allow those with identical numbers of SNPs to be visualised).

Fig 3 shows the number of SNPs and the time difference between disease onset in the prior contact and the case for the 62 pairs with ≤10 SNPs. From the slope of the linear regression the mutation rate is estimated at 0.33 SNPs/ year (95% CI 0.18–0.49, r2 24%, p<0.001).

The characteristics of the cases and prior smear positive contacts, and the associations with transmission, are shown in Table 1. Much the strongest association with transmission was the intensity of the contact (p<0.001). Information on intensity was missing for 16 pairs; 5 because questions on intensity were not asked in the first year of the study. Confirmed transmission was more common from family members (42.4%, 56/132) than from non relatives (19.4%, 6/31, p = 0.02), and especially from spouses and parents (61.1%). The proportion of confirmed transmissions decreased with increasing age of the index case and was lower in earlier years of the study.

**Table 1:** Number of confirmed transmissions by characteristics of the prior contact, the case, and the relationship between them among 163 case-prior contact pairs in which the prior contact had smear positive pulmonary tuberculosis.

Characteristic		n/N	%	P*
**Prior Contact**				
Age	<25	12/22	54.6	0.3
	25–34	21/61	34.4	
	35–44	15/38	39.5	
	45+	14/42	33.3	
Sex	Female	40/94	42.6	0.2
	Male	22/69	31.9	
Isoniazid	Resistant	5/11	45.5	0.6
	Sensitive	57/152	37.5	
Lineage	1	4/18	22.2	0.4
	2	3/9	33.3	
	3	11/24	45.8	
	4	44/112	39.3	
HIV status	HIV-	29/63	46.0	0.4
	HIV+ no ART	24/66	36.4	
	HIV+ ART	3/5	60.0	
**Index Case**				
Age	<25	17/28	60.7	0.04
	25–34	20/56	35.7	
	35–44	17/49	34.7	
	45+	8/30	26.7	
Sex	Female	38/97	39.2	0.7
	Male	24/66	36.4	
HIV status	HIV-	24/49	49.0	0.1
	HIV+ no ART	23/75	30.7	
	HIV+ ART	2/7	28.6	
Year	1997–2001	15/55	27.3	0.1
	2002–2006	32/74	43.2	
	2007–2010	15/34	44.1	
**Relationship**				
Relation	Spouse	11/18	61.1	0.01
	Parent	11/18	61.1	
	Child	1/6	16.7	
	Sibling	15/35	42.9	
	Other relation	18/55	32.7	
	Not related	6/31	19.4	
Intensity	Low	16/81	19.8	<0.001
	High	17/30	56.7	
	Nursing	24/36	66.7	
Interval	<1 year	20/44	45.5	0.1
	1–1.99 yrs	11/38	29.0	
	2–4.99 yrs	17/54	31.5	
	5+ yrs	14/27	51.9	

* from Χ^2^ tests or Fisher’s exact tests if numbers are small.

Since intensity of transmission was so strongly associated with confirmed transmission, the multivariable analysis was restricted to the 147 pairs with information on intensity. Relationship was strongly correlated with intensity of transmission (for example, no non-relatives had high or very high intensity (nursing) contacts), and after adjusting for intensity, relationship was no longer associated with transmission. After adjusting for intensity, the associations with age of the case and year of diagnosis of the case became stronger (Table 2), and there were borderline associations with sex of the case (higher in males) and age of the source contact (lower proportion confirmed from older contacts). None of the other factors were associated with transmission once adjusted for intensity and the other factors shown in Table 2. The adjusted odds ratio was 0.56 (95%CI 0.23–1.4) for HIV positive source contacts vs HIV negative source contacts, and 0.51 (0.19–1.4) for HIV positive index cases vs HIV negative index cases.

**Table 2:** Factors associated with confirmation of transmission in the multivariable logistic regression analysis among 147 case-prior contact pairs with information on intensity of contact.

	Univariable			Multivariable, adjusted for the other factors in the table		
	OR	95% CI	P*	OR	95% CI	P*
High intensity (vs low)	5.3	2.1–13.1		3.1	1.1–8.8	
Nursing (vs low)	8.1	3.4–19.6	<0.001	11.6	4.2–32.1	<0.001
Age case (per year)	0.96	0.93–0.99	0.003	0.94	0.90–0.98	0.002
Age contact (per year)	0.98	0.96–1.0	0.1	0.97	0.4–1.0	0.06
Male case (vs female)	0.89	0.46–1.7	0.7	2.2	0.92–5.3	0.07
Year case (per year)	1.1	0.99–1.2	0.07	1.1	1.0–1.3	0.04

OR = odds ratio

* From likelihood ratio test.

Table 3 shows estimates of the proportion of disease attributable to transmission from known contacts among patients with first episode culture-confirmed disease. It is assumed that the proportion of contacts with confirmed smear positive disease (59.7%) is the same in all groups, including those not identified in the database. Overall 9.4% of tuberculosis cases were attributable to transmission from known contacts. This was higher in younger individuals (11.8% in those aged <35) than older individuals (7.7% in those aged 35+), in women (10.9%) than men (7.9%), and in 2004–2010 (10.3%) than in the earlier years (7.7%). Family contacts were much more important as a source than other known contacts (87% of sources overall) and this was particularly marked in women (96% vs 78% in men).

**Table 3:** Estimate of the proportion of first episode culture-confirmed cases attributable to known smear positive contacts.

	A	B	C	D	
	Proportion with contact (from data)	Proportion with smear +ve contact (Ax0.597)	Proportion smear +ve transmitting(from data)	PAF (BxC)	PAF for any contact
Overall					
Family	32.1%	19.2%	42.4%	8.1%	
Other^*^	10.9%	6.5%	19.4%	1.3%	9.4%
Age < 35					
Family	35.1%	21.0%	47.1%	9.9%	
Other	11.1%	6.6%	28.6%	1.9%	11.8%
Age 35+					
Family	31.4%	18.8%	37.1%	7.0%	
Other	10.6%	6.3%	11.8%	0.7%	7.7%
Female					
Family	39.6%	23.7%	44.6%	10.5%	
Other	9.4%	5.6%	7.1%	0.4%	10.9%
Male					
Family	26.5%	15.9%	38.8%	6.1%	
Other	10.0%	6.0%	29.4%	1.8%	7.9%
1997–2003					
Family	33.6%	20.1%	28.6%	5.7%	
Other	14.0%	8.4%	23.1%	1.9%	7.7%
2004–2010					
Family	32.7%	19.6%	50.0%	9.8%	
Other	6.2%	3.7%	13.3%	0.5%	10.3%

*The proportion with other contacts excludes those with family contacts as well

PAF = population attributable fraction.

## Discussion

This is the first study to calculate the proportion of tuberculosis attributable to transmission from known contacts established through whole genome sequencing. This technique provides the most accurate method available of verifying the source of transmission. We confirm the expected strong association of transmission with intensity of contact and smear positivity, but estimate that, overall, known smear positive prior contacts account for less than 10% of tuberculosis cases in this community, and that even for those with a prior contact with smear positive tuberculosis in their family, there was a >50% chance that they acquired their tuberculosis elsewhere.

The results are consistent with our earlier findings and those from elsewhere based on RFLP,[5,8,10,20] and a low proportion of tuberculosis attributable to household transmission has also been reported in other high prevalence settings using other techniques.[21] Not surprisingly, a higher proportion of transmissions are confirmed in low prevalence settings.[6,7,12,13,22] Using SNPs is a more accurate measure of similarity than RFLP, and the cut-off we used is in line with that used in other studies. The mutation rate in our study was also similar that found elsewhere measured between or within patients.[12,13] Using genomic similarity to confirm transmission assumes that neither the initial nor the subsequent episode is due to a mixed infection. We have previously found a low proportion of mixed infections in this setting, of around 3%,[15] and using whole genome sequencing, 1.3% of samples had levels of heterogeneity suggesting mixed infection.[18] We have assumed that the proportion of confirmed transmissions we found in the case-contact pairs for which we had samples and sequence available is applicable to all such pairs. Although cases had a higher chance of being included in the analysis if they named more contacts, there was no evidence that including those with multiple contacts lowered the proportion confirmed: those naming more contacts had a slightly higher proportion of confirmed transmissions.

The calculations of the population attributable fraction make the additional assumption that the proportion of contacts with smear positive disease overall in this population is the same as the proportion in the contacts we identified in the database. This is probably an overestimate: many of those not identified may not have had tuberculosis at all. We have not included smear negative cases because in our data there was no confirmed transmission so they would not have contributed to the PAF; however, this was based on only 17 pairs. We have only included contacts known to and identified by the tuberculosis patients. This will underestimate the true number of contacts: some will be undiagnosed and others not known to or remembered by the index cases. Not knowing a contact is more likely for less close contacts, who only contribute a small proportion of transmissions. Arbitrarily doubling the number of “other contacts” in those with no known family contact, for example, would only increase the proportion of tuberculosis attributable to known contacts from 9.4% to 10.6%.

As well as variation by type of contact in the probability of a transmission being confirmed, we found variation by age and time period. The decreased proportion of confirmed transmissions to older cases is consistent with a higher proportion of reactivation disease with increasing age. And the higher proportion of confirmed transmissions in recent years is consistent with lower tuberculosis incidence and reduced transmission in the community.[23,24] There were also differences by sex, with non-family contacts being relatively more important for men, who spend more of their time away from home. We found only weak evidence of reduced transmission from (smear positive) HIV positive source contacts compared to HIV negative source contacts; given the high prevalence of HIV infection among tuberculosis patients (about 60% of smear positive patients in this population at the time of the study), HIV-infected patients are an important source of *M*.*tuberculosis* transmission.

### Conclusions

In this setting, where tuberculosis is endemic, almost half of the individuals with culture-confirmed tuberculosis have had identified contact with previous patients with tuberculosis, often in their close family. Yet even those with a family contact with smear positive tuberculosis are likely to have acquired their tuberculosis elsewhere, and close contacts contribute less than 10% of sources of tuberculosis in the population. Where a high proportion of smear positive tuberculosis patients are HIV positive they may be a major source of *M*.*tuberculosis* transmission.
